# Change of the upper airway after mandibular setback surgery in patients with mandibular prognathism and anterior open bite

**DOI:** 10.1186/s40902-019-0230-4

**Published:** 2019-11-26

**Authors:** Kyungjin Lee, Soon Jung Hwang

**Affiliations:** 1 Department of Oral and Maxillofacial Surgery, School of Dentistry, Seoul National University, Seoul, Republic of Korea; 2 HSJ Dental Clinic for Oral and Maxillofacial Surgery, Wannam Building 2,3F, 349 Gangnam-daero, Seocho-gu, Seoul, 06626 Republic of Korea

**Keywords:** Mandibular setback, Pharyngeal airway space, Mandibular prognathism, Open-bite, Hyoid bone

## Abstract

**Purpose:**

It has been reported before that the amount of pharyngeal airway space (PAS) significantly decreases following mandibular setback (MS) surgery in patients with mandibular prognathism (MP). Further, MP patients with an anterior open-bite (AOB) presentation may show a larger decrease in PAS compared with those without AOB. However, studies on postoperative PAS changes in MP patients with AOB remain rare. This study sought to evaluate changes in PAS and hyoid bone positioning following MS surgery in MP patients with and without AOB.

**Patients and methods:**

Twenty patients who underwent two jaw surgery involving MS movement were included. Patients were divided into a non-AOB group (*n* = 10; overbite > 2 mm) and an AOB group (*n* = 10; overbite < − 4 mm). Three-dimensional changes in PAS and hyoid bone positioning were compared and statistically evaluated pre- and postoperatively using computed tomography (CT).

**Results:**

The mean magnitude of MS was 6.0 ± 2.8 mm and 5.6 ± 3.2 mm in the non-AOB group and AOB group, respectively. The oropharyngeal volume and upper hypopharyngeal volume were significantly reduced after surgery in both the groups (*p* = 0.006 and *p* = 0.003), while the retroglossal cross-sectional area was significantly reduced only in the AOB group (*p* = 0.028). Although the AOB group showed a larger decrease in PAS, the difference was not statistically significant between the groups. The position of the hyoid bone showed significant posterior and inferior displacement only in the AOB group, while the vertical displacement of the hyoid bone showed a statistically significant difference between the two groups.

**Conclusion:**

PAS was significantly decreased after MS in both the groups, while only the AOB group presented a statistically significant reduction in the retroglossal cross-sectional area. Vertical displacement of the hyoid bone showed a statistically significant difference between the groups, while the PAS change was not. Surgeons should be aware of potential postoperative airway problems that may arise when performing MS surgeries.

## Introduction

Mandibular setback (MS) surgery is usually performed for aesthetic and functional correction in patients with mandibular prognathism (MP). Because MS involves backward movement of the mandible along with muscle and other soft tissue attachments, the pharyngeal airway space (PAS) and positioning of the tongue are inevitably affected [1]. One of the main concerns of these consequences is their potential to cause obstructive sleep apnea (OSA) [2]. OSA is known to increase the risk of hypertension, ischemic myocardial diseases, and cerebral vascular diseases and thus is considered potentially life-threatening [3].

Earlier studies on MS and PAS changes were performed using lateral cephalometric analysis. These studies repeatedly showed that the PAS decreased after MS surgery [1, 4–6], and the degree of PAS reduction was smaller with bimaxillary surgery than with mandible-only surgery [5]. In contrast, in certain cases, particularly in patients with AOB, the joint movement of the mandible to maxilla could scale down the reduction of the PAS. However, cephalometric study for the analysis of change in the PAS is limited to two-dimensional measurements on the sagittal plane, while the PAS is a three-dimensional (3D) structure that requires 3D volume and cross-sectional analysis for proper assessment [7, 8]. Some studies using computed tomography (CT) reported no decrease in PAS [9] or even increase in the total PAS after bimaxillary surgery involving posterior movement of the mandible [10, 11], but the majority of them confirmed reduced oropharyngeal space, cross-sectional area posterior to the tongue and soft palate, or decreased lateral and frontal dimensions of the PAS [7, 8, 10–14]. It has also been speculated that MS may induce OSA [5, 7]. A recent study by Yang et al. [14] found that four of twelve patients who underwent orthognathic surgery involving substantial MS presented with new-onset OSA in the postoperative period.

Patients with MP and AOB commonly present with a higher mandibular plane angle, higher gonial angle, forward tongue position, and macroglossia [15, 16]. Anterior tongue placement in patients with AOB is regarded as a physiological adaptation [15, 17]. Moreover, patients with AOB have more constricted upper airways [18, 19] and inferiorly positioned hyoid bones [20, 21]. Partial glossectomy is often warranted when posterior movement of the mandible is planned [16, 22]. Given the fact that patients with AOB have a higher ratio of tongue volume to oral space volume [15], it is rational to speculate that the PAS can become constricted following backward movement of the anteriorly positioned and enlarged tongue after surgical closure of AOB when compared with those without AOB. Nevertheless, studies on postoperative PAS changes in MP patients with AOB are only rarely reported.

The purpose of this study was to evaluate the changes in PAS and hyoid bone positioning after MS using 3D facial multislice CT scans and to compare the results between patients with and without AOB.

## Patients and methods

### Patients

This study was conducted under the Institutional Review Board approval from Seoul National University Dental Hospital (CRI12036). The study procedure was performed in accordance with Helsinki Declaration revised in 2008. This study included 20 patients with skeletal class III malocclusion who underwent bilateral sagittal split ramus osteotomy (BSSRO) and Le Fort I osteotomy with or without genioplasty at the Department of Oral and Maxillofacial Surgery, Seoul National University Dental Hospital in Seoul, Korea. All patients showed < 10 mm of MS at the B point and < 4 mm of maxillary movement at the A point in the immediate postoperative lateral cephalogram. Patients were divided into two groups: an AOB group (*n* = 10) with < − 4 mm of preoperative overbite and a non-AOB group (*n* = 10) with an overbite of > 2 mm. Those who underwent BSSRO alone were excluded, and all surgeries were performed by a single surgeon. In all patients, multislice CT scans were taken 1 month before (T1) and 3–6 months after the surgery (T2). The mean age of the patients at the time of surgery was 20.1 ± 6.1 years (range 16–26 years) and the sex ratio was 11:9 (male:female). All patients received pre- and postoperative orthodontic treatment, and semirigid fixation was completed using four-hole titanium miniplates and screws.

### Cephalometric analysis

To measure surgical changes and the degree of overbite, the lateral cephalograms of each patient were taken in the maximum intercuspal position at a magnification ratio of 1.1:1 prior to surgery (T0) and immediately after surgery (T1). Each cephalogram was traced on an acetate paper. Cephalometric analysis was conducted using the superimposition technique. Reference points included the sella (S), nasion (N), A point (A), B point (B), upper incision (U1), and lower incision (L1). All reference points were transferred to the lateral cephalograms taken at T1. An *x*- and *y*-coordinate system was established, wherein the *x*-axis was constructed by rotating S–N clockwise by 7° (SN7), while the *y*-axis was constructed by drawing a line through the sella, perpendicular to SN7 (SN7v). The linear parameters consisted of the *x* and *y* coordinates of A and B.

### Evaluation of pharyngeal airway in CT

3D facial multislice CT scans were obtained from all patients using a CT scanner (SOMATOM sensation 10; Siemens, Munich, Germany) with the following parameters: 120 kVp, 80 mAs, and a slice thickness of 0.75 mm. Digital image files were saved in the Digital Imaging and Communications in Medicine format and imported into the Invivo Dental software program (Anatomage, San Jose, CA, USA). CT images were rendered into volumetric images. The reconstructed sagittal, axial, and coronal slices and 3D images were then obtained. To standardize the measurements and minimize errors, the Frankfort horizontal (FH) plane was constructed to reorient the 3D images. The FH plane was constructed from the right and left porions and the right orbitale.

The airway study in CT consisted of three components: (1) changes in distance and area in axial CT, (2) changes in airway volume in 3D images, and (3) changes in hyoid bone positioning.

To assess the linear distance and cross-sectional area of the posterior airway, pre- and postoperative measurements were collected at two different levels, specifically at the retropalatal level (the level of the most posterior point of the soft palate parallel to the FH plane) and the retroglossal level (the level of the most posterior point of the tongue base parallel to the FH plane). For both levels, the largest mesiodistal width (L_MD_), anteroposterior length (L_AP_), and cross-sectional area (S) were measured.

**Fig. 1:**
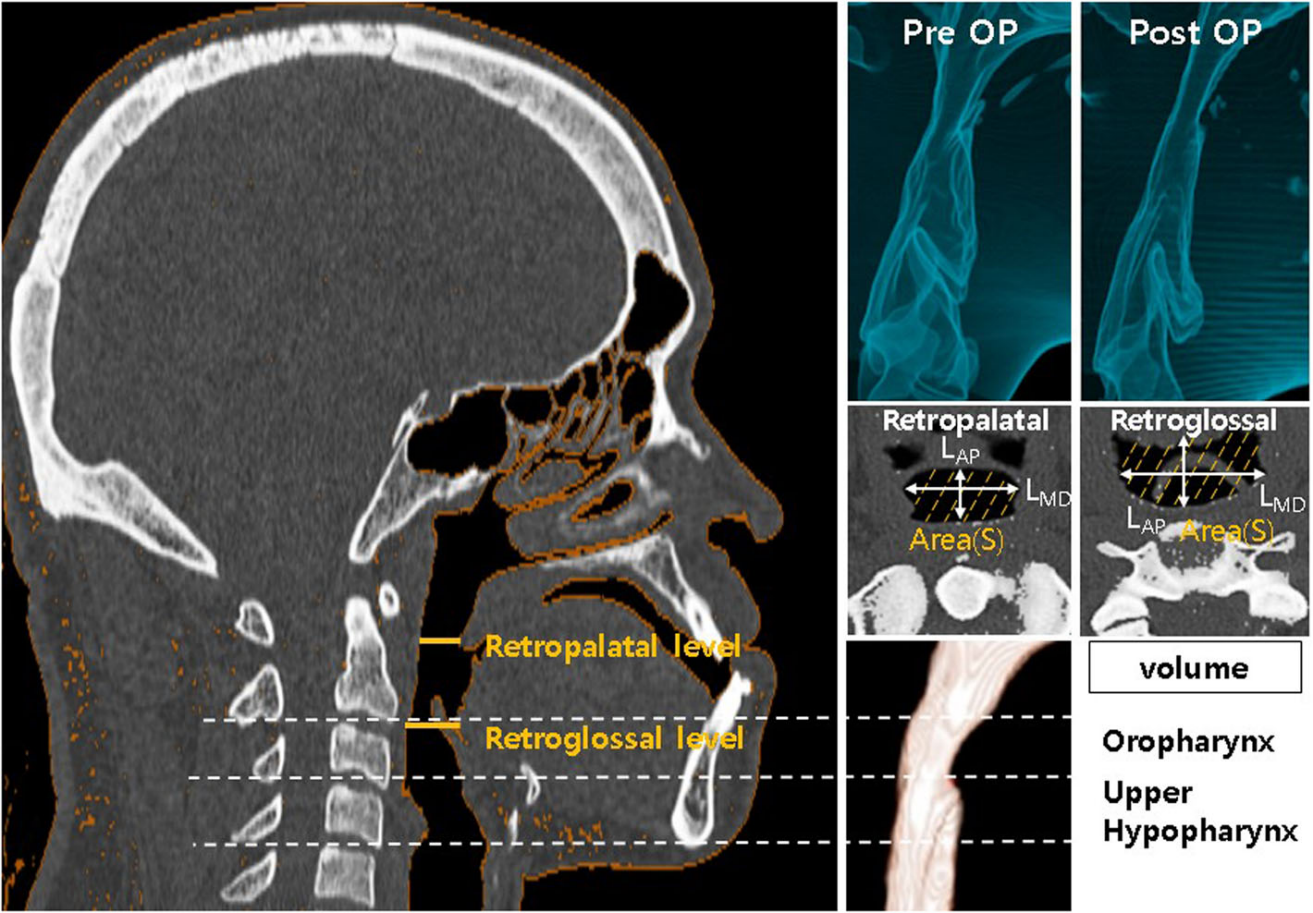
Measurements in volume, linear distance, and area in the pharyngeal airway. The largest transverse width, anteroposterior length, and cross-sectional area were measured at the level of the most constricted point of the soft palate and tongue base. The airway was divided as follows, and each volume was measured for the oropharyngeal area (between CV1 line and CV2 line) and the upper hypopharyngeal area (between CV2 line and CV3 line), with the CVn lines being parallel to the FH plane

To evaluate the airway volume, a range of − 1024 to − 600 Hounsfield units was set as the threshold value of the CT image, where the pharyngeal airway could be effectively differentiated from the neighboring soft tissue. The pharyngeal airway was divided into the following two regions using cervical vertebra (CV) reference points, with the CVn plane defined as the plane parallel to the FH plane passing through the most inferior point of the CVn: (1) the oropharyngeal volume between CV1 and CV2 planes (V_o_) and (2) the upper hypopharyngeal volume between the CV2 and CV3 planes (V_h_). The pharyngeal airway volumes of all patients were measured by the same examiner using the Invivo Dental software program (Anatomage, San Jose, CA, USA), and postoperative volume changes were calculated (Fig. 1).

**Fig.2:**
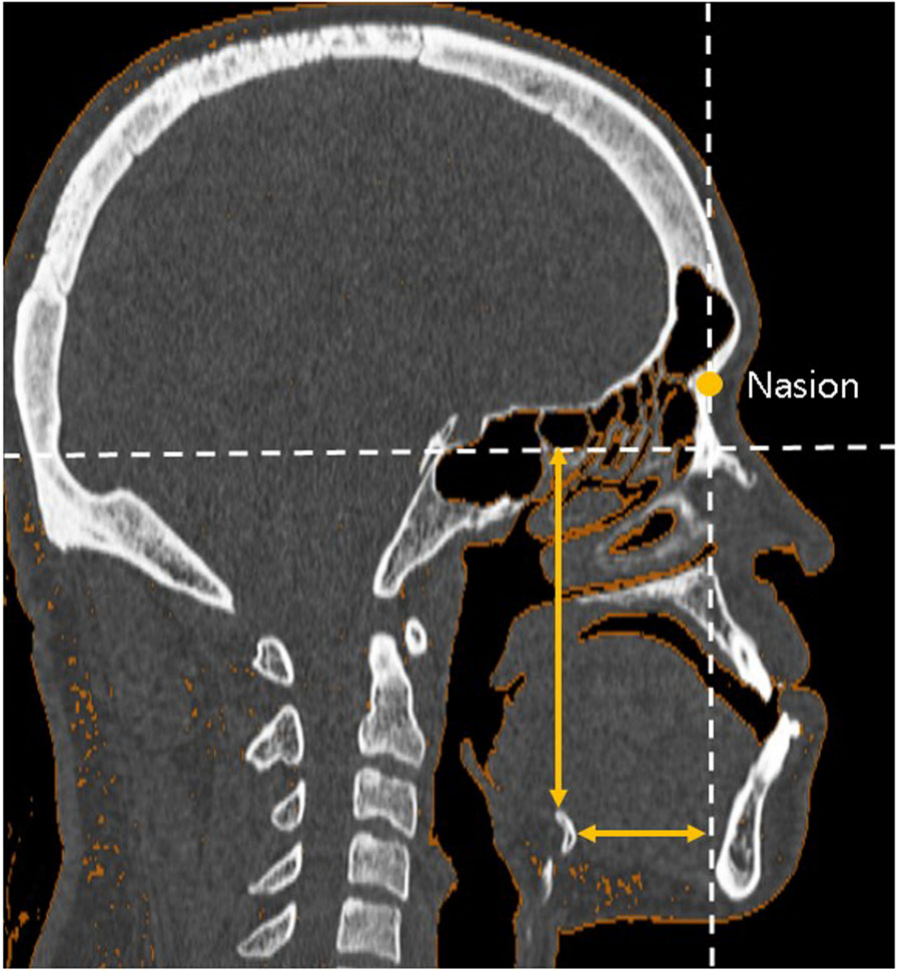
Vertical and horizontal positioning of the hyoid bone was measured in CT (mm). The *x*-axis was defined as the line tangent to the inferior point of the sella turcica and parallel to the FH plane, while the *y*-axis was defined as the line passing through the nasion and perpendicular to the *x*-axis

To evaluate the hyoid bone positioning before and after surgery, two reference lines were defined. The *x*-axis consisted of the line tangent to the inferior portion of the sella turcica and parallel to the FH plane, while the *y*-axis consisted of the line perpendicular to the *x*-axis and passing through nasion. The horizontal and vertical positions of the hyoid bone were measured using the distances between the most anterosuperior point of hyoid bone to the *x*-axis (H_x_) and *y*-axis (H_y_), respectively (Fig. 2).

### Statistical analysis

The descriptive statistics of the preoperative and postoperative measurements were processed using the SPSS for Windows version 21 software program (IBM Corp., Armonk, NY, USA). Kolmogorov–Smirnov analysis was performed for all measured parameters attested to the normal distribution of values. The preoperative and postoperative cephalometric measurements and pharyngeal airway volume, length, and area were analyzed using the Wilcoxon signed-rank test. A paired *t* test was performed to compare the differences between the groups. Differences were considered to be significant at *p* < 0.05

## Results

### Surgical changes of maxilla and mandible

On average, the mandible was moved backward by 6.0 ± 2.76 mm and upward by 1.3 ± 1.6 mm in the non-AOB group and backward by 5.6 ± 3.2 mm and upward by 5.4 ± 2.2 mm in the AOB group at the B point. Furthermore, the maxilla was moved forward by 1.4 ± 2.2 mm and upward by 1.9 ± 2.3 mm in the non-AOB group and forward by 2.0 ± 2.0 mm and upward by 3.9 ± 1.7 mm in the AOB group at the A point (Table 1.)

**Table 1:** Surgical movements following two jaw surgery for mandibular setback movement

		Surgical movement	
		non-AOB	AOB
Mandible	Horizontal	− 5.95 ± 2.67	− 5.55 ± 3.24
	Vertical	1.25 ± 1.57	5.35 ± 2.17
Maxilla	Horizontal	1.35 ± 2.19	1.95 ± 1.96
	Vertical	1.90 ± 2.32	3.90 ± 1.66

### Pharyngeal airway

**Fig. 3:**
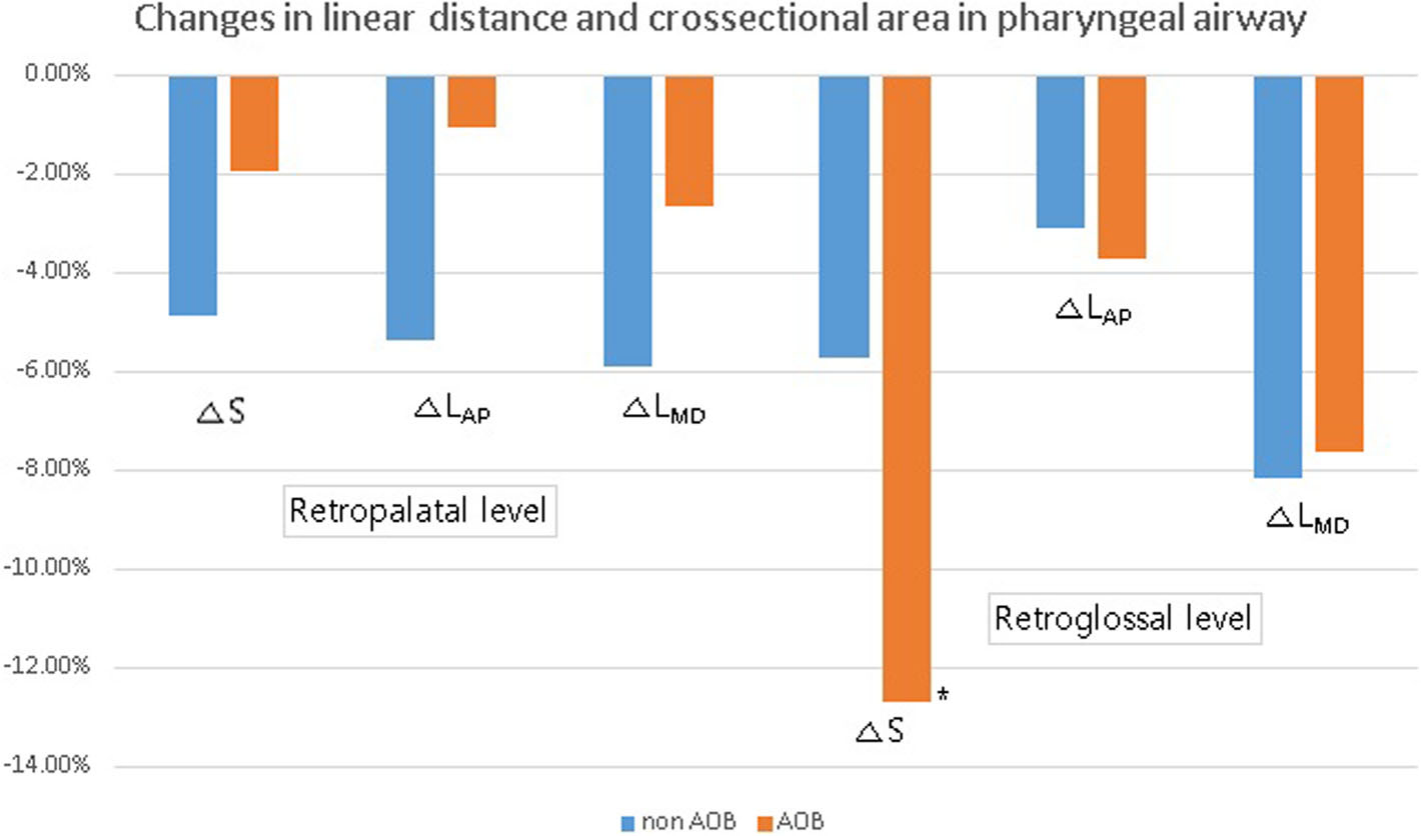
The average change in the pharyngeal airway volume. The postoperative airway volume was significantly decreased in both the groups (*p = 0.006 and *p = 0.003), even though the difference between the two groups was not statistically significant

The postoperative airway volume was significantly decreased in both the oropharynx and upper hypopharynx in both the groups (*p* < 0.05). The average change in the airway volume was − 12.16% ± 28.18% in the non-AOB group and − 14.62% ± 10.60% in the AOB group in the oropharynx and − 14.71% ± 20.02% in the non-AOB group and − 17.43% ± 20.18% in the AOB group in the upper hypopharynx, respectively. Although there was a greater reduction of PAS in the AOB group in both the oropharynx and upper hypopharynx, the difference was not statistically significant (Table 2; Fig. 3).

**Table 2:** Results of pharyngeal airway and hyoid bone position measurements

	Pre-op	Post-op	Change
Non-AOB			
Volume			
V_o_ (mm3)	5661.4 ± 1356.5	4991.8 ± 1908.4	− 12.16 ± 28.19%*
V_h_ (mm3)	4962.3 ± 2086.4	4115.6 ± 1704.5	− 14.71 ± 20.02%*
Retropalatal level			
L_AP_ (mm)	7.4 ± 2.2	7.7 ± 3.5	− 5.37 ± 17.69%
L_MD_ (mm)	20.1 ± 3.8	19.0 ± 5.2	− 5.91 ± 13.05%
S (mm2)	132.7 ± 64.1	129.6 ± 84.4	− 4.87 ± 36.02%
Retroglossal level			
L_AP_ (mm)	10.7 ± 2.1	10.5 ± 3.4	− 3.12 ± 17.96%
L_MD_ (mm)	29.1 ± 3.47	26.7 ± 4.0	− 8.15 ± 8.48%
S (mm2)	250.5 ± 54.8	242.0 ± 97.5	− 5.74 ± 24.73%
Hyoid bone			
Horizontal	43.9 ± 14.3	46.0 ± 15.8	5.45 ± 10.46%
Vertical	91.4 ± 28.5	91.7 ± 29.8	0.32 ± 4.44%^#^
AOB			
Volume			
V_o_ (mm3)	5354.2 ± 2611.8	4367.7 ± 1703.9	− 14.62 ± 10.61%*
V_h_ (mm3)	4693.5 ± 2171.8	3853.9 ± 2231.6	− 17.43 ± 20.19%*
Retropalatal level			
L_AP_ (mm)	7.7 ± 2.4	8.2 ± 2.6	− 1.07 ± 15.66%
L_MD_ (mm)	22.6 ± 6.9	21.7 ± 7.1	− 2.67 ± 19.85%
S (mm2)	170.8 ± 79.4	166.3 ± 90.1	− 1.95 ± 31.21%
Retroglossal level			
L_AP_ (mm)	12.0 ± 4.2	11.3 ± 3.5	− 3.72 ± 15.09%
L_MD_ (mm)	28.8 ± 2.7	26.5 ± 3.8	− 7.64 ± 11.70%
S (mm2)	290.6 ± 122.0	252.5 ± 104.7	− 12.68 ± 14.97%*
Hyoid bone			
Horizontal	42.1 ± 16.5	46.7 ± 17.8	12.77 ± 9.61%*
Vertical	91.1 ± 31.7	95.1 ± 32.7	4.4 ± 5.11%*^#^

*p < 0.05 in Wilcoxon singed-rank tests

^#^*p* < 0.05 in paired *t* test

**Fig. 4:**
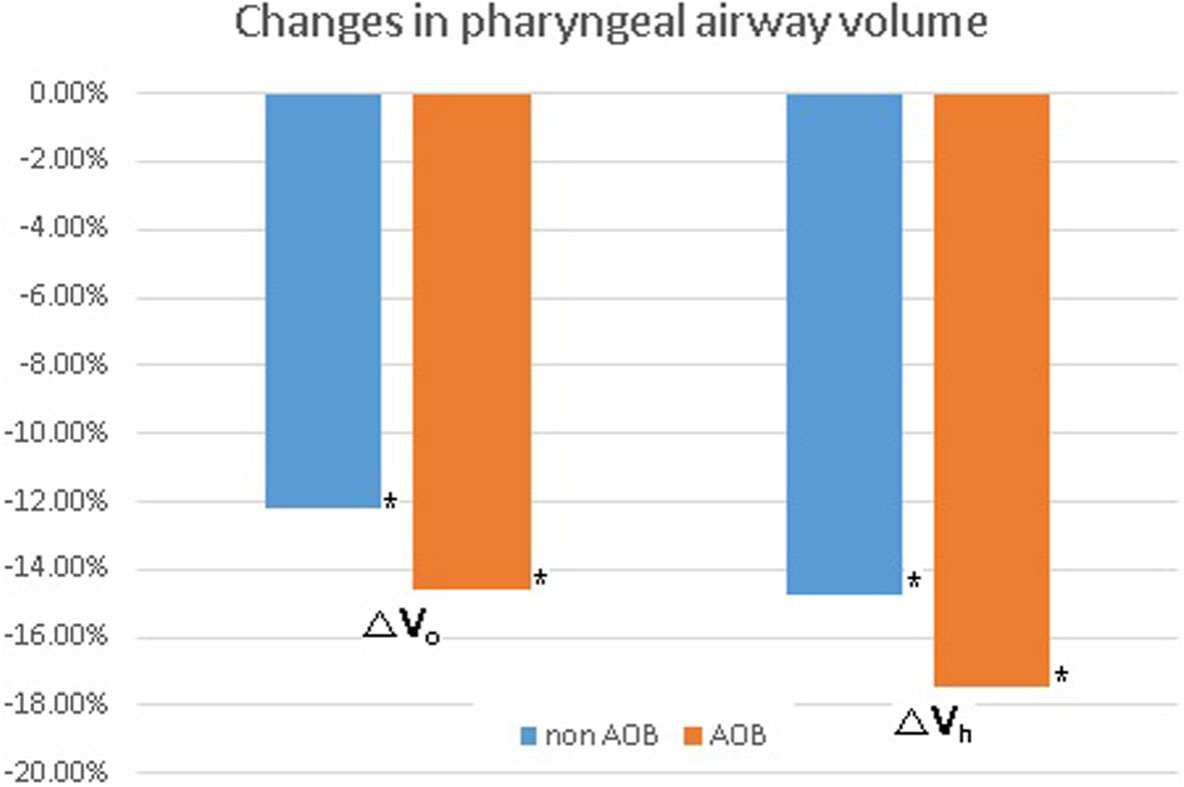
The average changes in linear distance and cross-sectional area in the pharyngeal airway. All parameters were decreased postoperatively in the retropalatal level and retroglossal level, while S at the retroglossal level was significantly decreased in the AOB group (*p = 0.028)

The linear distance and area were also decreased after surgery in both the groups. At both the retropalatal and retroglossal levels, L_MD_, L_AP_, and S decreased, but there was no statistically significant difference in these changes except for S at the retroglossal level in the AOB group (*p* = 0.03). Intergroup analysis showed no significant difference present in all parameters at both levels (Table 2; Fig. 4).

### Hyoid bone positioning

**Fig. 5:**
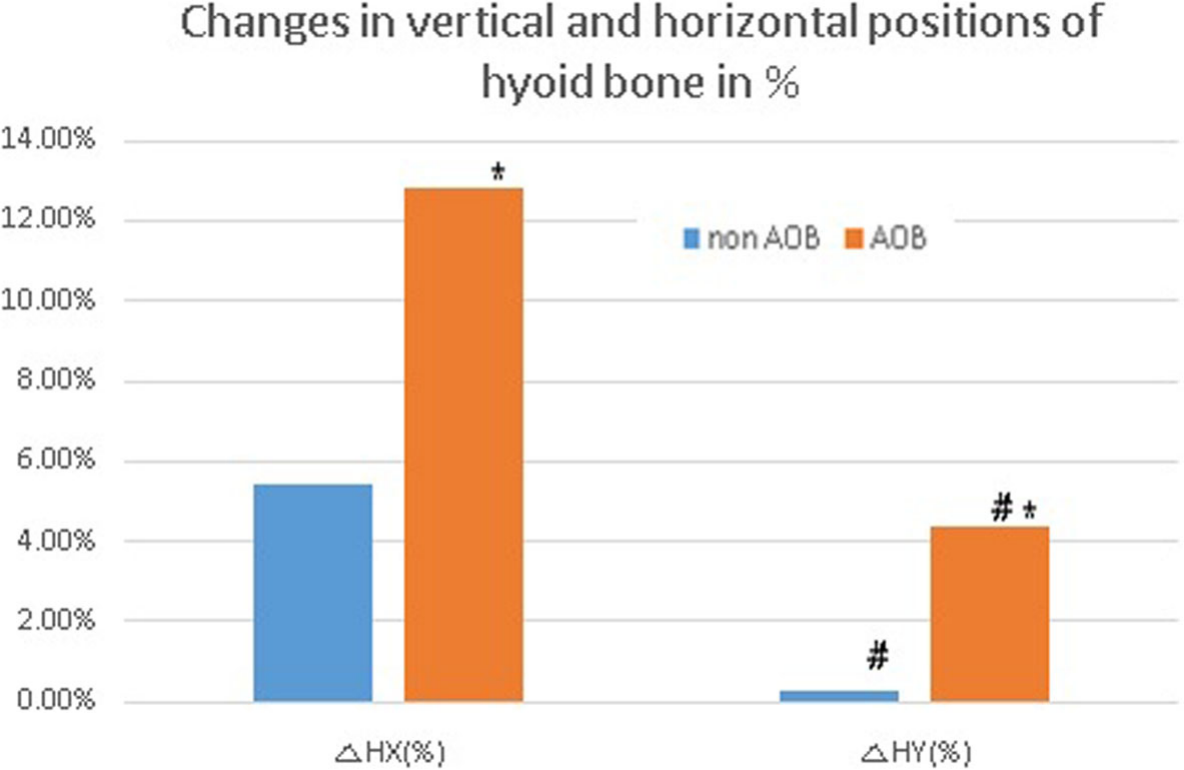
Vertical and horizontal changes of the hyoid bone location. The hyoid bone was displaced backward and downward in both the groups. Vertical and horizontal changes in the hyoid bone positioning in the AOB group showed statistical significance (*p = 0.022 and *p = 0.028), and there was a statistically significant difference between the two groups in terms of vertical displacement of the hyoid bone (^#^*p* = 0.02)

The hyoid bone was displaced backward by 2.02 ± 5.13 mm and downward by 0.32 ± 2.93 mm in the non-AOB group and backward by 4.63 ± 4.80 mm and upward by 4.02 ± 4.40 mm in the AOB group (*p* > 0.05). Vertical and horizontal changes in the hyoid bone in the AOB group demonstrated statistical significance (*p* < 0.05) (Fig. 5). There was a statistically significant difference between the two groups in terms of vertical displacement of the hyoid bone (*p* = 0.02).

## Discussion

Changes in the airway space after MS surgery have gained attention in recent decades and have been widely studied, yet the outcomes vary among studies. Some of the earlier studies did not report any significant changes being present in the airway space after MS [9, 23, 24]. Several other studies performed using CT, however, suggested that PAS was significantly decreased after MS [2, 3, 10–13, 25–27]. For example, Lee et al. achieved a decrease of 14.07% in the total volume following a mean MS of 9.20 mm [11], while Kim et al. reported a decrease of 15.80% in total PAS and decreases of 22.08%, 8.10%, and 12.43% in the oropharynx, nasopharynx, and hypopharynx, respectively, after a mean MS of 8.25 mm [28]. Elsewhere, Yang et al. showed a decrease of 22.30% in the oropharynx and of 25.24% in the hypopharynx after a mean MS of 10.53 mm [14]. In the present study with mean MS values of 6.0 mm in the non-AOB group and 5.6 mm in the AOB group, the PAS was significantly decreased in both the groups at the oropharynx (12.10% and 14.62% in the non-AOB and AOB groups, respectively) and upper hypopharynx (14.71% and 17.42% in the non-AOB and AOB groups, respectively).

Cases of AOB present common skeletal characteristics including a negative overbite, augmented anterior facial height, steep mandibular plane, and increased gonial angle [29–31]. Patients with AOB also have different characteristics of the airway, hyoid bone, and tongue. Chang et al. and Cho et al. reported that the tongue was positioned more anteriorly in MP patients with AOB [32, 33], and Lee et al. showed that the hyoid bone was positioned more inferiorly in MP patients with AOB [21]. Tarkar et al. determined in their study that the PAS was significantly narrower and the position of the dorsum of the tongue was significantly higher in AOB patients compared with non-AOB patients [19], while Abu Allhaija et al. showed that the anteroposterior dimension of PAS was narrower in AOB patients than in non-AOB patients [34]. These features are unfavorable for comfortable respiration and may influence the changes in PAS after MS surgery. However, studies on postoperative PAS changes in MP patients with AOB are hard to find in the literature. According to the results of the present study, there was a greater reduction in the oropharyngeal and upper hypopharyngeal spaces and the sectional space area at the tongue base level in the AOB group, but the difference was not statistically significant. Further studies with larger sample sizes are warranted to elucidate differences in PAS changes between the two groups.

The hyoid bone is a movable hard tissue suspended via muscles and ligaments connecting it to the skull base, mandible, pharynx, and tongue. Thus, its position is easily influenced by the movement of surrounding tissues [35, 36]. A positional assessment of the hyoid bone is used to evaluate the physiological equilibrium of the suprahyoid muscles, infrahyoid muscles, and tissues surrounding the hyoid bone [6, 37]. Both posterior and inferior displacements of the hyoid bone have been generally noted after MS surgery [26, 38]. Such movements are believed to be the result of soft tissue (e.g., muscle) adaptation [26, 39], and collectively represent one of the most important factors in the phenomenon of PAS narrowing. PAS narrowing is a very important clinical consequence because it can cause upper airway crowding and collapse [3]. In the present study, the hyoid bone showed posterior and inferior displacement in both the groups, which is in accordance with the majority of findings of previous studies. However, the magnitude of vertical and horizontal displacement showed statistically significant differences only in the AOB group. In the intergroup comparison, only the vertical displacement of the hyoid bone showed a statistically significant difference.

## Conclusion

Following MS surgery in MP patients, oropharyngeal and upper hypopharyngeal airway volume were significantly decreased in both the AOB and non-AOB groups. Meanwhile, the hyoid bone showed significant inferior and posterior displacement in the AOB group only. In a comparison between the two groups, the hyoid bone showed a significantly larger inferior displacement in the AOB group. Although the difference was not statistically significant, the magnitude of PAS reduction was larger in the AOB group in both the oropharynx and upper hypopharynx. The results of this study suggest that the possibility of the onset of a respiratory disturbance such as OSA following MS may be greater in MP patients with AOB than in those without AOB. Therefore, surgeons should be aware of potential postoperative airway problems when performing MS surgeries.

## Data Availability

All data generated or analyzed during this study are included in this article and its supplementary information files.

## Abbreviations

2D: Two-dimensional
3D: Three-dimensional
AOB: Anterior open-bite
BSSRO: Bilateral sagittal split ramus osteotomy
CSA: Cross-sectional area
CT: Computed tomography
CV: Cervical vertebra
MP: Mandibular prognathism
MS: Mandibular setback
OSA: Obstructive sleep apnea
PAS: Pharyngeal airway space
